# Effect of psychological treatment on attentional bias in eating disorders

**DOI:** 10.1002/eat.20500

**Published:** 2008-05

**Authors:** Roz Shafran, Michelle Lee, Zafra Cooper, Robert L Palmer, Christopher G Fairburn

**Affiliations:** 1 Department of Psychiatry, Oxford UniversityOxford, United Kingdom; 2 University of Leicester, Branoon Mental Health Unit, Leicester General HospitalLeicester, United Kingdom

**Keywords:** cognitive therapy, attentional bias, eating disorders, dot probe

## Abstract

**Objective:**

The aims of these studies were (a) to investigate the relationship between attentional bias and eating disorders and (b) examine the impact of psychological treatment on attentional bias.

**Method:**

The first study compared performance on a pictorial dot probe of 82 female patients with clinical eating disorders and 44 healthy female controls. The second study compared the performance of 31 patients with eating disorder on the same task before and after receiving 20 weeks of standardized cognitive behavior therapy. Twenty-four patients with eating disorder served as wait-list controls

**Results:**

With the exception of neutral shape stimuli, attentional biases for eating, shape, and weight stimuli were greater in the patient sample than the healthy controls. The second study found that attentional biases significantly reduced after active treatment only.

**Conclusion:**

Attentional biases may be an expression of the eating disorder. The question of whether such biases warrant specific intervention requires further investigation. © 2008 by Wiley Periodicals, Inc. Int J Eat Disord 2008

## Introduction

The question of the role of attentional biases in psychopathology is an intriguing and important one. It has been suggested that biases such as selective attention toward threatening words or pictures have a causal link to anxiety.^1^ Such suggestions are supported by a seminal study demonstrating that experimentally inducing differential attentional responses to emotional stimuli using a modified dot-probe task influences emotional vulnerability assessed by a stress task.^2^ It remains to be seen, however, whether such specific attentional training is necessary for improvement in symptoms. Data from studies of the impact of psychological treatment on attentional biases indicate that focusing specifically on these biases in treatment is not necessary to reduce them.^3^,^4^ Some studies, however, report no changes in attentional bias with treatment, e.g., in post-traumatic stress disorder^5^ whereas others report a partial decrease in such biases with treatment (e.g., in social phobia^6^) or ambiguous results.^7^

There are also contradictory findings in the literature with regard to the association between change in attentional bias and change in psychopathology. While some studies have found that change in a specific symptom is associated with changes in emotional processing,^8^ a number have reported a lack of association between change in psychopathology and change in the attentional bias.^5^,^7^ These null findings cast doubt on the clinical utility of the Stroop task as a measure of clinical outcome. Lack of specificity of findings in studies using eating-disorder salient stimuli (positive findings have also been noted in young female samples without eating disorders) also points to the limited clinical utility of this assessment method and other measures, such as the dot-probe task are recognized as better measures of selective attention.^9^ The dot-probe task has recently been adapted to use pictures rather than words to enhance its clinical relevance.^10^,^11^

A pictorial dot-probe task has recently been used in a study of patients with eating disorders and anxious and nonclinical controls.^12^ This study found that patients with eating disorders showed attentional biases for eating and weight stimuli over and above that shown by controls. Such findings replicated and extended previous information on attentional biases in patients with eating disorders using the dot-probe task with words.^13^

Based on the previous pictorial dot-probe study,^12^ it was concluded that selective attention to eating-disorder relevant information exists, is specific to patients with eating disorders and can be assessed using the modified dot-probe task. The study was the first to include patients with ‘‘eating disorder not otherwise specified (EDNOS),’’ which is particularly important given that the majority of patients who present to clinics have this diagnosis.^14^ However, the study also raised some questions, the most obvious of which concerned the lack of attentional bias for shape relevant stimuli, the inability of the task to determine the precise nature of the attentional bias, and the relatively modest associations between the core psychopathology of eating disorders and attentional bias. In addition, the study did not address the question of whether these biases were modified by a treatment that did not include a specific focus on attentional bias toward eating, shape, and weight.

The overall goal of the present research was to establish whether patients with eating disorders show attentional biases for eating, shape, and weight-related stimuli and, if so, whether such biases change with effective treatment. Two studies were conducted. The first investigated the presence of such biases in a large group of patients with eating disorders and healthy controls. The second examined whether such biases changed in patients treated with cognitive behavioral therapy. Both studies received ethical approval from the local University Ethics Committee.

## Study 1

The aim of Study 1 was to replicate the previous research on biases in eating disorders with a larger sample.^12^

## Method

### Participants

The patient sample comprised 82 female patients recruited for a transdiagnostic eating disorder treatment trial and were referred by local clinicians. Patients were randomized to either focus on their core eating disorder psychopathology or on another maintaining mechanism (clinical perfectionism, core low self-esteem, interpersonal problems, or mood intolerance). Male patients were excluded from the study given the nature of the stimuli used (female bodies), and the fact that previous research has used female samples. Each patient completed the dot-probe task immediately prior to starting treatment. The group comprised 50 patients with ED-NOS [including six with binge eating disorder (BED)], 27 with bulimia nervosa (BN), and five with anorexia nervosa (AN). These women were compared with 44 female aged-matched healthy controls from the local community, with no current depression and no current or past history of an eating disorder.

### Materials

#### Stimuli

The stimuli used in the dot-probe task were the same as those used in a previous study^12^ and comprised positive, negative, and neutral ‘‘eating’’ and ‘‘body shape’’ pictures and neutral ‘‘body weight’’ pictures (referred to as the ‘‘target image’’) paired with a control ‘‘animal’’ picture matched for emotional valence (participants were screened for animal phobias).

#### Modified Dot-Probe Task

The dot-probe task used here has been described in a previous study.^12^ Target and control images were presented alongside each other on a computer screen. These were then replaced with a probe (X) which appeared in a location corresponding to the center of one of the two pictures. Participants were required to indicate the position of the probe (i.e., left or right). The position of the eating-disorder relevant image and the position of the probe were balanced across trials.

### Measures

#### Eating Disorder Examination—Self-Report Version (EDEQ^15^)

This self-report measure assesses eating disorder features over the last 28 days and is based on the eating disorder examination (EDE^16^). The questionnaire has good reliability and validity.^17^

### Data Analysis

The data analysis was based on reaction times (RTs) for correct responses (see Table 1). Following protocols adopted in previous studies,^12^ latencies of less than 200 ms and more than 2,000 ms were excluded and outliers were removed by excluding detection latencies that were beyond two standard deviations from their mean (i.e., from each individual’s mean RT across all stimuli). A bias score was calculated^18^(i.e., RT when target and probe were in different positions—RT when target and probe were in the same position). Vigilance toward a target image was indicated by a positive bias score and avoidance away from a target by a negative bias score.

**TABLE 1:** Age, body mass index, eating disorder psychopathology, and accuracy scores (and SDs) for patients with eating disorder and control groups

	Eating Disorder (*n* = 82)	Controls (*n* = 44)	*t, p*
Age	25.87 (6.92)	26.41 (6.50)	*t*(120) = 0.42, *p*>.05
BMI	21.59 (4.12)	23.09 (3.92)	*t*(122) = 1.67, *p* = .051
EDE-Q restraint**	3.57 (1.41)	1.34 (1.06)	*t*(120) = 12.05, *p*<.001
EDE-Q eating concern**	2.67 (1.44)	0.66 (0.84)	*t*(120) = 11.54, *p*<.001
EDE-Q shape concern**	3.85 (1.28)	1.66 (1.12)	*t*(120) = 15.59, *p*<.001
EDE-Q weight concern**	3.63 (1.26)	1.23 (1.02)	*t*(120) = 11.06, *p*<.001
% Correct responses on the dot-probe task*	98.24 (5.30)	99.98 (0.15)	*t*(124) = 2.17, *p*<.05

* *p*<.05.

** *p*<.005.

## Results

### Nature of the Sample

Demographic and eating disorder psychopathology scores are presented in Table 1 along with error scores on the dot-probe task. Groups were comparable on age and differed significantly on all EDE-Q scales.

### Bias within Patients

Differences in RTs for eating and shape stimuli were investigated via 3 (valence; positive, negative, neutral stimuli) × 2 (probe position; same as target, opposite to target) repeated measures ANOVAs. As there were only neutral weight stimuli, the effect of probe position was investigated via a paired-sample *t*-test.

#### Eating stimuli

There was a significant valence × probe position interaction (*F*(2,80) = 44.04, *p* < .001). Patients were significantly quicker to respond to the probe when it was in the same location as negative eating images (*t*(81) = 5.68, *p* < .001) but significantly slower to respond to the probe when it was in the same location as the positive eating images (*t*(81) = 8.13, *p* < .001). No difference was found in RTs for neutral eating stimuli (*t*(81) = 0.69, *p* > .05). These results remained statistically significant when controlling for BMI.

#### Weight stimuli

Patients were significantly quicker to respond to the probe when it was in the same location as the weight images (*t*(81) = 5.68, *p* < .001).

#### Shape stimuli

There was a significant valence × probe position interaction (*F*(2,80) = 9.67, *p* < .001). For negative shape stimuli, participants were significantly quicker to respond to the probe when it was in the same location as the target picture than when it appeared in the opposite location (mean RTs = 569.77 and 659.74 ms, respectively; *t*(81) = 4.47, *p* < .001) and this was also the case for neutral shape stimuli (mean RTs = 569.82 and 604.13 ms, respectively; *t*(81) = 2.22, *p* < .05). However, RTs were not affected by probe position (mean RTs = 592.69 and 593.43 ms, respectively; *t*(81) = 0.44, *p* > .05). This pattern of results remained when controlling for BMI.

Further analyses (which are not reported here) indicated that these results were comparable across all initial eating disorder diagnoses.

### Patients vs. Controls

Bias scores across groups are presented in Table 2.

**TABLE 2:** Mean bias scores in all patients with eating disorder (before treatment) and controls (and SDs)

Stimuli Type	Patients (*n* = 82)	Controls (*n* = 44)	*t, p*
Eating stimuli			
Positive**	−68.65 (109.41)	−3.34 (44.66)	*t*(124) = 3.78, *p* <.001
Negative**	110.70 (123.34)	−11.69 (50.95)	*t*(124) = 6.29, *p* <.001
Neutral	10.35 (135.21)	−4.45 (95.22)	*t*(124) = 0.65, p >.05
Shape stimuli			
Positive	0.74 (152.42)	−12.00 (62.29)	*t*(124) = 0.60, p >.05
Negative*	89.97 (182.22)	4.91 (76.54)	*t*(124) = 2.96, *p* <.005
Neutral	34.31 (139.78)	−0.21 (58.41)	*t*(124) = 1.56, p >.05
Weight stimuli			
Neutral**	100.15 (157.88)	−21.19 (65.61)	*t*(124) = 4.87, *p* <.001

* *p* <.005.

** *p* <.001

1. Bias for eating stimuli across groups. There was a significant valence by group interaction (*F*(2,123) = 24.49, *p* < .001). Patients with eating disorders had greater bias scores than the controls for positive and negative eating stimuli but not neutral eating stimuli.
2. Bias for shape stimuli across groups. There was a marginally significant valence by group interaction (*F*(2,123) = 3.08, *p* = 0.05). Patients with eating disorders showed significantly greater bias than controls for negative shape stimuli but not positive or neutral shape stimuli.
3. Bias for weight stimuli across groups. Bias scores for weight stimuli were significantly higher in patients with eating disorders than controls.

## Discussion

Study 1 involved a replication of our previous study^12^ with a larger sample. As predicted and as was the case with our previous study,^12^ patients with eating disorders were faster to react to negative eating and neutral weight stimuli and slower to react to positive eating stimuli. However, unlike the previous study and more consistent with theoretical predictions, participants in this study were significantly quicker to respond to the probe when it was in the same location as the target picture for negative and neutral shape pictures (for example, images of large thighs, or images of elbows). No bias was found for positive shape stimuli (e.g., slim figures). It may be the case that the pictures of shape need to be personally relevant to detect such biases and the positive images used did not have meaning for the participants. These biases were greater in patients with eating disorders than healthy controls with the exception of the bias for positive and neutral shape stimuli.

## Study 2

The aim of Study 2 was to examine the effect of treatment on attentional biases in eating disorders. Three hypotheses were proposed for patients with eating disorders.

1. Bias scores will change after treatment, and;
2. Such changes will not be attributable to practice effects (i.e., bias scores will not change simply as a function of doing the task twice).
3. The degree of change in bias after treatment will be associated with the degree of change in core psychopathology of the eating disorder after treatment.

## Method

### Participants

Hypothesis 1a (regarding change in biases with treatment) and hypothesis 2 (regarding the relationship between change in psychopathology and change in bias scores) used a subset of 31 of the original sample of 82 patients. All the 31 patients had been assigned to immediately receive 20 weeks of treatment for their eating disorder. This sample comprised 18 patients with ED-NOS (including six with BED), and 13 patients with BN. (Patients who were significantly underweight (BMI \ 17.5) were excluded from this sample as their treatment lasted 40 weeks.) Participants were asked to complete the dot-probe task immediately prior to starting treatment, and then again at the end of treatment. Mean body mass index for this sub-sample was 22.72 (SD = 4.24) and the mean age was 26.03 years (SD = 6.94). The mean EDE-Q scores pre-and post-treatment are provided in Table 3.

**TABLE 3:** EDE-Q scores and interference (bias) scores (ms) for patients with eating disorder before and after treatment (*n* = 31)

Before Treatment	After Treatment	*t, p*	
EDE-Q scores			
Restraint*	3.21 (1.63)	1.06 (1.38)	*t*(29) = 5.76, *p* <.001
Eating concern*	2.68 (1.40)	1.01 (1.19)	*t*(29) = 10.46, *p* <.001
Shape concern*	3.69 (1.21)	2.31 (1.25)	*t*(29) = 12.67, *p* <.001
Weight concern*	3.55 (1.28)	1.95 (1.48)	*t*(29) = 8.44, *p* <.001
Interference scores			
Eating stimuli			
Positive*	−45.51 (95.44)	26.18 (105.17)	*t*(30) = 3.23, *p* <.005
Negative*	130.27 (117.70)	−2.65 (106.07)	*t*(30) = 4.23, *p* <.005
Neutral	−6.22 (143.85)	−7.94 (121.18)	*t*(30) = 1.27, *p* >.05
Shape stimuli**			
Positive	−40.37 (97.64)	11.18 (97.85)	
Negative	66.70 (137.79)	13.35 (88.28)	
Neutral	20.90 (89.56)	38.07 (118.21)	
Weight stimuli			
Neutral*	76.43 (100.14)	27.49 (59.38)	*t*(30) = 3.29, *p* <.005

* Scores before and after treatment are significantly different (at least *p* <.05).

** Post hoc *t*-tests were not computed as the initial ANOVA was nonsignificant.

To control for any change in bias being due to having done the task twice (hypothesis 1b), a subset of 24 patients from the original pool of 82 patients in Study 1 who had been randomly assigned to a wait list condition within the treatment trial were asked to complete the task at the start and end of their delay period of 8 weeks. The 8-week delay period was chosen for ethical reasons (i.e., not wishing to prolong the time before treatment). This sample comprised 15 patients with ED-NOS (none of whom had BED), six patients with BN and three patients with AN. (None of these 24 patients were also included in the sample of 31 patients used to address hypotheses 1a or 2). Mean body mass index for this sample was 21.75 (SD = 3.90) and the mean age was 25.35 years (SD = 6.25). Mean EDE-Q subscale scores were as follows: Restraint = 3.88 (SD = 1.09), eating concern = 2.50 (SD = 1.27), shape concern = 3.75 (SD = 1.67), and weight concern = 3.52 (SD = 1.22).

### Procedure

Participants were asked to complete the dot-probe task (as described in Study 1) prior to starting treatment (as already described for Study 1), and immediately after completing 20 weeks of ‘‘enhanced’’ cognitive-behavioral treatment (*n* = 31).^19^ This treatment does not use specific attentional training techniques to address attentional biases but is an improved version of the therapy used by Fairburn et al.^20^ It is suitable for the full range of clinical eating disorders. Those in the delayed treatment condition were also asked to complete the dot-probe task immediately before and after the delay. Patients were assessed using the eating disorder examination questionnaire (EDE-Q^15^). Outliers and bias scores were as described for Study 1.

#### Pre-and Post-Treatment EDE-Q Scores

Pre and post EDE-Q scores are presented in Table 3. All EDE-Q scores were significantly reduced after treatment.

*Hypothesis 1a*. Bias scores will change after treatment.

Bias scores for patients before and after treatment are presented in Table 3.

##### Eating stimuli

There was a significant time (2) × valence (3) interaction for bias scores controlling for BMI scores prior to and after treatment (*F*(2,28) = 6.43, *p* < .005) indicating that treatment resulted in changes for interference scores. Paired t-tests indicated that bias scores for positive and negative eating stimuli reduced significantly after treatment (*t*(30) = 3.23 and *t*(30) = 4.23, *p* < .005, respectively). However, no change was found for neutral eating stimuli (*t*(30) = 1.27, *p* >.05).

##### Shape stimuli

There was no significant time (2) × valence (3) interaction for bias scores controlling for BMI scores prior to and after treatment (*F*(2,28) = 0.68, *p* >.05) therefore further follow up tests were not carried out.

##### Weight stimuli

Bias scores for neutral weight stimuli reduced significantly after treatment (*t*(30) = 3.29, *p* < .005).

Hypothesis 1b. Such changes will not be attributable to practice effects (i.e., bias scores will not change simply as a function of doing the task twice).

Bias scores in the 24 patients completing the task at the start and end of their wait list delay are presented in Table 4. A series of paired t-tests indicated that bias scores did not alter as a function of doing the task twice, for any of the stimuli used (see Table 4).

**TABLE 4:** Interference (bias) scores (ms) for patients with eating disorder at two points prior to treatment (*n* = 24)

Stimuli Type	Time 1	Time 2	*t, p*
Eating stimuli			
Positive	−52.27 (90.43)	−102.37 (126.99)	*t*(22) = 1.51, *p*>.05
Negative	171.05 (198.09)	111.92 (138.50)	*t*(22) = 1.56, *p*>.05
Neutral	−89.04 (164.54)	−9.62 (139.27)	*t*(22) = 0.84, *p*>.05
Shape stimuli			
Positive	57.01 (163.43)	90.06 (198.41)	*t*(22) = 1.03, *p*>.05
Negative	147.39 (116.97)	103.26 (126.10)	*t*(22) = 1.00, *p*>.05
Neutral	52.26 (137.57)	46.26 (122.18)	*t*(22) = 0.16, *p*>.05
Weight stimuli			
Neutral	65.66 (142.88)	104.44 (100.46)	*t*(22) = 1.51, *p*>.05

*Hypothesis 2*. The degree of change in bias after treatment will be associated with the degree of change in core psychopathology of the eating disorder after treatment.

Pearson correlation coefficients were calculated in patients receiving 20 weeks of treatment (*n* = 31) to assess the association between change in eating disorder psychopathology after 20 weeks treatment (i.e., change on EDE-Q Global scores and all other EDE-Q scales) and change in bias scores after 20 weeks of treatment for all types of stimuli. A moderate but significant positive correlation was found between changes on EDE-Q Global scores and changes on bias for negative shape stimuli (*r* = 0.41, *p* < .01), but not changes on bias for other stimuli. A significant correlation was found between changes in scores on the EDE-Q Shape Concern scale and changes in bias for negative shape stimuli (*r* =0.33, *p* < .05), but not changes in bias for any other stimuli. Changes on the EDE-Q Eating Concern scale were significantly correlated with and changes in bias for negative eating stimuli, neutral eating stimuli, and negative shape stimuli (*r* = −.40, −.42, and −.50, respectively). The greater the reduction in eating psychopathology, the greater the reduction in biases for eating-disorder related stimuli. With the exception of the correlation between change in Eating Concern scores and change in bias for negative shape stimuli, all correlations remained significant when controlling for change in BMI.

## Discussion

This study found that attentional biases in patients with eating disorders reduce with treatment and that such changes cannot be attributed to practice effects. However, although the design allowed for the control of doing the task twice, the wait list period of 8 weeks is different from the 20-week active period of therapy. It cannot be ruled out that with a further 12 weeks, there would have been significant change in the biases of those in the wait list condition.

This reduction in biases is consistent with the data from studies of generalized anxiety disorder.^3^ The implication is that efforts to change such processing biases directly by developing training tasks may not generally be necessary. In the cognitive-behavioral treatment received by patients, self-focused attention to disliked body parts and sensations were addressed if they were prominent, but no specific attentional training intervention was used. However, the data reported are group means, and it is likely that for some individuals, the biases did not fully remit. For such individuals, it may be the case that such biases are themselves acting as a barrier to symptom change and an additional intervention to address the biases directly could be helpful.

The finding that such biases normalize after treatment suggests that they may be an expression of the eating disorder. If this was the case, then the degree of change in bias should have been associated with the degree of change in the symptoms of the eating disorder. However, although some modest associations were found, on the whole there was no close relationship between the amount of change in the bias and change in symptoms of the eating disorder. Why, then, do the biases change? It is possible that treatment changes the way that people process information regarding eating, shape, and weight regardless of change in the behavioral symptoms of the eating disorder. It is also possible that the measures of information processing biases and eating psychopathology are too different to be able to accurately assess the relationship between the two.

## Conclusion

Together the two studies demonstrate the presence of specific attentional biases in eating disorders, and indicate that such biases improve with cogni-tive-behavioral treatment. They have a number of limitations. First, the valence of the stimuli used were those rated by people without eating psychopathology. Although this is the norm in research of this type in anxiety disorders and depression, it is nevertheless a limitation. Second, no active treatment comparison group was included to establish whether the change in biases is specific to cognitive-behavioral therapy (which does include self-focused attention) or whether it occurs with a treatment such as interpersonal psychotherapy in which attention to symptoms of the eating disorder is not addressed at all. Third, the time period of the waiting list group was shorter than that of the active treatment group. Despite these difficulties, this is the first study to examine the change in such biases with treatment using the dot-probe task, it included a wait-list control and it included patients with the full range of eating disorders. Furthermore, the patients were diagnosed using standardized measures of psychopathology and biases were assessed using a state-of-the-art pictorial dot-probe task.

In conclusion, the study used a pictorial dot-probe task to demonstrate that attentional biases in patients with eating disorders (a) exist and (b) normalize after treatment. Further investigation of the nature of the attentional biases and their connection to eating disorder psychopathology is warranted.

The participants were recruited for a Trust-funded treatment experiment (046386). The authors are grateful to the following people for their assistance with data collection: Marianne O’Connor, Caroline Adams, Elizabeth Payne, Jocasta Webb, Clare Nollett, and Jackie Wales.
